# Frontiers in human factors: embedding specialists in multi-disciplinary efforts to improve healthcare

**DOI:** 10.1093/intqhc/mzaa108

**Published:** 2020-09-09

**Authors:** Ken Catchpole, Paul Bowie, Sarah Fouquet, Joy Rivera, Sue Hignett

**Affiliations:** Department of Anesthesia and Perioperative Medicine, Medical University of South Carolina, Charleston, SC, USA; NHS Education for Scotland, Institute of Health and Wellbeing, University of Glasgow G12 8RZ, UK; Improvement Institue, Children’s Mercy Hospital, Kansas City, MO, USA; Patient Relations & Spiritual Services, Froedtert Hospital, Milwaukee, WI, USA; Loughborough Design School, Loughborough University LE11 3TU, UK

**Keywords:** Human factors, patient safety, human error, quality improvement, implementation

## Abstract

Despite the application of a huge range of human factors (HF) principles in a growing range of care contexts, there is much more that could be done to realize this expertise for patient benefit, staff well-being and organizational performance. Healthcare has struggled to embrace system safety approaches, misapplied or misinterpreted others, and has stuck to a range of outdated and potentially counter-productive myths even has safety science has developed. One consequence of these persistent misunderstandings is that few opportunities exist in clinical settings for qualified HF professionals. Instead, HF has been applied by clinicians and others, to highly variable degrees—sometimes great success, but frequently in limited and sometimes counter-productive ways. Meanwhile, HF professionals have struggled to make a meaningful impact on frontline care and have had little career structure or support. However, in the last few years, embedded clinical HF practitioners have begun to have considerable success that are now being supported and amplified by professional networks. The recent coronavirus disease of 2019 (COVID-19) experiences confirm this. Closer collaboration between healthcare and HF professionals will result in significant and ultimately beneficial changes to both professions and clinical care.

## Introduction

Human Factors (HF) perspectives are familiar to many in healthcare. Thousands of people are now benefiting from the value those perspectives can bring to a multitude of care contexts. HF has been applied in acute care [1], primary care [2, 3], emergency care [4], and home care [5]. It has influenced device design [6], health IT [7], health system design [8], architecture [9], simulation [10], education [11, 12], quality improvement [13], safety [14] and reliability [15]. Studies have analyzed systems of work [16], teamwork [17], decision-making [18], displays [19], device interactions [20], risks [21], threats [22], performance shaping factors [23], environmental and organizational approaches [24, 25] and regulatory influences [26]. The methods of direct observation, interviews, task analysis, usability studies, human reliability analysis and incident analysis have been frequently, reliability and creatively applied. However, there is much more that could be done to realize the full potential of HF principles and expertise for patient benefit, staff well-being and organizational performance [27]. This has raised a number of questions about how HF knowledge and expertise could be integrated better into clinical work.

Prior to the application in healthcare, HF knowledge and expertise had arisen mostly from either consumer products and software, or heavily engineered industries [28]. In these industries, human actions are often carried out through the technology, with the HF focus on integrating humans, processes and technologies into a ‘top-down’ work system with known inputs, goals and constraints. Clinical systems are not necessarily like those other industries, though there remains a need design for human use. Healthcare is more complex, with multiple different goals and outcomes, a huge variation in inputs, work contexts, procedures and necessary variation in process (to account for individual patient needs). New procedures and technologies are continually being introduced. It was never purposely engineered but has grown organically over thousands of years to meet a range of human needs. Every healthcare organization is different, and procedures can vary from provider to provider and patient to patient. How the system works is opaque, and it can be difficult to understand what is, or what should be, happening. The particular, and arguably unique, constraints and demands in healthcare mean that while the principles of system safety science in general, and HF in particular, are similar, their application and mechanisms of effect can be very different.

While healthcare also contains many different groups who have a strong interest in improving human work (e.g. HR professionals, organizational development specialists, clinical risk, patient safety and quality improvement advisors), the HF professional can provide much-needed ‘added-value’ to multi-disciplinary team efforts to jointly optimize overall organizational performance and human well-being. This paper explores the application of HF in relation to those that apply it; what we need to do to be more effective; how this might fundamentally change both healthcare and HF professions and the opportunities for service transformation it might present.

## Stubborn Myths

Despite much progress in the application of systems thinking to clinical work, there remains a focus on ‘error’ as a clinical-professional issue to be ‘fixed’ rather than signifying on the deeper causes of error, within the clinical system. Instead, the psychology, engineering, sociology and other expertise necessary to address the deep systems problems associated with accidental harms has often been simplified to lookalike principles that sadly missed important details [29]. What might be termed, pseudo-HF approaches have arisen [30, 31] where system-level thinking is eschewed for well-meaning but limited training, or other attempts at direct behavioral change that apply HF tools but do not consider systems complexity.

The HF profession focuses on integrating humans and systems and brings with it knowledge and experience of a range of concepts, principles, standards and methods. These are arguably better suited to understanding and resolving many of the problems and issues routinely experienced in highly complex, dynamic healthcare systems Consequently, HF professionals can spend significant amounts of time and energy challenging the myths and misunderstandings, which continue to prevail, particularly amongst key groups and leaders, and trying to persuade some to unlearn what they think they know about Human Factors Engineering (HFE). Among the widely held and stubbornly persistent myths are:

- The myth of the individual practitioner. Instead of clinical outcomes being driven by a single clinician, they are driven by the collaboration of many individuals across a wide number of disciplines. This myth is perpetuated in, for example, physician league tables, despite clear evidence around the importance of teamwork, and organizations in outcomes [32].
- The myth of the perfect system. When harm happens, the tendency is to believe that this was an exception in an otherwise safe system. However, extensive observational evidence demonstrates that clinical systems are far from perfect [33] and require a wide range of work-arounds and trade-offs every day to be successful [34].
- The myth that reliable care processes lead to safe system safety in complex care situations is an emergent property of the whole system, while reliability is a property of individual system components. Every system component can function exactly as intended (reliability), but the complex interactions between components can lead to an unsafe system. [35].
- The myth of ‘human error.’ As people are always implicated in accidents, there has been an assumption that ‘removing’ human error (or humans), would prevent harm. Again, multiple safety scientists demonstrate that this is a simplistic view of accident causation as it fails to acknowledge the everyday trade-offs humans in the system are required to make. Indeed, it is the ability for humans to vary what they do that is responsible for everyday effective systems function.
- The myth of standardization and control of variation. A further perturbation of these myths is the need to control individual human variation, and the ubiquitous call for ‘standardization.’ However, given that every patient is different, every unit, every specialty and every organization is also different, variation is not only required, but is an essential part of patient-centered care.
- The ‘Zero’ myth. Though ‘zero’ harms (‘never events’) may be useful goals, they are, by their nature, unattainable [29]. Instead, risks, costs and the number of patients we treat need to be traded against each other. A system where zero harm could be achieved would have such a huge financial burden and would have such a profound effect on care delivery that it would fail in a range of other ways. The risk/benefit considerations and outcomes in response to the recent COIVD-19 pandemic are an excellent example of how finances and treatment needs need to be carefully balanced with risks to patients and staff, often in the absence of clear data.
- The myth of linear determinism. A further fallacious assumption is that process and outcome are directly related; thus, a good outcome means everything went well (and no further attention is needed), while bad outcome must mean something went wrong, which requires investigation. Again, safety science demonstrates the inadequacy of these assumptions. Many things can and do go wrong in the care of a patient, but few affect outcomes, because the people in the system are always adapting to moment-to-moment demands in ways that technologies, policies and processes do not.

Instead of understanding healthcare systems as complex and adaptive, where trade-offs judgements are being made between safety, cost and throughput all the time [36], the pervading myths have led to the rigid application of approach or tools regardless of the context. The use of quality processes derived from highly engineered systems such as Failure Modes and Effects Analysis (FMEA) and lean/6 sigma suit some contexts and not others, and at least has required adaptation for healthcare [13, 37]. Checklists are a complex socio-technical intervention requiring understanding of the tasks, risks, design and use, so evaluating a checklist only on patient outcomes does not help understand the task, team, organizational or clinical specialty variations necessary for it to be adopted and effective [38, 39]. Crew resource management or non-technical skills training is often delivered in the absence of appropriate organizational support, and without a sufficient understanding of the underlying engineering that made those approaches successful in aviation. There is no doubt that all are valuable but fail when applied without consideration of the wider systems in which they will be used. Learning from other industries benefits from translation, based on mechanism-of-effect and differing constraints. This is where HF expertise can help.

Seeing HF as a set of ‘tools’ that can be ‘applied’ in a rule-based way, or trained in short course will have narrow benefits and vastly under-appreciates the value that the discipline can bring. HF principles can be applied across highly diverse contexts, organizations and units, informing both improvement in complex systems, and the methods to hypothesize and evaluate them. The expertise of HF is in understanding the potential effects of interventions, and the selection of the appropriate evaluation from a wide range of methods. Some clinicians who practice HF have embraced this more nuanced understanding of HF; some have not; and a few have been deeply resistant to this broader systems perspective. We need to think carefully about how to enhance the enthusiasm for HF, while challenging the range of misperceptions that limit its value. The use of HF tools by clinicians is an excellent place to start and should be encouraged and supported with higher level HF. Then, we need to find ways in which HF professionals and clinicians can work together to bring the right HF principles to bear in the right place, at the right time.

## Embedding Human Factors Professionals in Healthcare

Despite a growing recognition of the need and value of HF in clinical work across the world, one consequence of the persistent misunderstanding of system safety science is that few opportunities exist in clinical settings for qualified HF professionals, limiting the availability of appropriately sophisticated HF expertise [27]. Until the United States Food and Drug Administration (FDA) mandated HF as part of device design in 2011 [40], HF healthcare work was funded through research, with much of it based at universities rather than hospitals, on a by-project basis rather than being applied full time alongside and in support of clinicians. Given that return on investment can be difficult to calculate and effect on outcomes difficult to measure in a non-linear system, a direct business case is still hard to make. However, this did not prevent the expansion of quality improvement and patient specialists, who are mostly unqualified in their subject matter area, and lack appropriate professional development and regulation, unlike their HF counterparts. While the FDA regulatory demands have vastly increased the number of HF experts within the healthcare industry, and there is much that could be learned from this about the development of and spread of HF expertise, device design is often far removed from clinical practice. Professional healthcare HF practice outside of device development has therefore been disparate, ad hoc and precarious, with no formal career structure.

These professional and financial constraints have also limited the ability of HF professionals to understand ‘work as done,’ the complexity of healthcare systems, or the new language and approaches required to address clinical needs. This has created a chicken-and-egg problem, where HF professionals have not been employed in care organizations, because there has been a limited understanding of what they can do, no clear and immediate application, no business case, no clear evidence base and without day-to-day frontline experience of work as done they have not always been effective. A ‘simple checklist’ or ‘training’ are easier to package and demonstrate value, no matter how simplistic (and occasionally thoroughly erroneous [41]), than a multitude of more appropriate approaches to improvement in complex adaptive systems [42]. This is a significant barrier to progress, but it has begun to change. In the last few years, hospitals across the world have begun to employ clinically embedded HF professionals—that is, fully qualified HF practitioners who work alongside clinicians every day to apply HF methods and perspectives. Countries that have embedded HF practitioners include the USA, Canada, Australia, the UK and Portugal and most likely many others. Positions have also been appearing in in regulatory roles.

Effective applied clinical practice thus requires both the technical demands of understanding when and how to apply HF approaches and the understanding of the complexity of clinical context of a given application. Healthcare organizations need to move beyond the underlying fixation on human errors and a lack of systems thinking. They need to know how they can employ HFE specialists and up skill key parts of the workforce through accredited routes at comparative low cost. They also need to know what sort of HF expertise to employ, and when. While there is formal route for HF professional accreditation (through professional societies such as Human Factors and Ergonomics Society (HFES), Chartered Institute for Ergonomics and Human Factors (CIEHF), International Ergonomics Association (IEA)), few clinicians, patient safety administrators or quality improvement specialists who practice have any formal qualifications, training or appropriate CPD around risk/safety/HFE when compared with many of their equivalents in other industrial sectors. Clinicians who champion HF causes would benefit from appropriate accreditation and access to and wider support from qualified HF professionals. They may also benefit from understanding the limitations of and adaptions for popular but limited paradigms such as checklists, teamwork training or non-technical skills. For example, effective teamwork skills in cardiac surgery are likely to be very different from the teamwork skills in robotic surgery [43]. Surgeons or anesthesiologists are often well placed to lead this type of approach, but do not always appreciate how these skills fit into the broader clinical systems picture. Broader and equally powerful principles—such as safety-II, user-centered design, task analysis, selection and training, controls and displays, situational awareness, anthropometry—need to be applied in different ways to solve new problems and/or translate to a new context. They are also continually updating as our theoretical and methodological knowledge improves.

In order to be effective at a local level, expertise needs to distributed rather than centralized. Rather than working independently, we need to support the application of HF locally with national and international networks. Often, clinicians and embedded HF practitioners work alone, or in small groups, often on the same problems as others in other organizations (for example, on Central Line Associated Blood Stream Infection (CLABSI), or Failure-to-Rescue, or Retained Foreign Objects). There may be common themes, but solutions might look very different in different organizations, or even across different units. The ability to share learning and best practices, many of which will not end up in clinical publications, could have a powerful effect in spreading—and especially translating—good practices for different work contexts. Such a network would also support embedded HF professionals who may be otherwise isolated from their peers and from new safety science, practice or methodological development.

There have also been a number of successful organizational approaches to support both clinicians applying HF and HF professionals working in healthcare. Most notably the Clinical Human Factors Group (www.chfg.org) that originally worked to introduce aviation-style approaches but has subsequently begun to embrace the wider systems view. Meanwhile, the UK Chartered Institute (www.ciehf.org) has been increasingly bringing together clinicians and professionals work on specific projects and has defined a set of skills, competence levels and training appropriate for clinical practice. In the USA, the Human Factors and Ergonomics Society (www.hfes.org) runs an annual conference devoted to healthcare HFs, while also supporting a specific healthcare technical group (www.hctg.hfes.org). Meanwhile, the Human Factors Transforming Health Network (HFTH) (www.hfthnetwork.org) has been developed specifically to support embedded clinical HF practice, through a network of practitioners, clinical champions and organizations aiming to deploy professional HF expertise. We anticipate increasing alignment of professional HF and clinical HF groups as the two fields continue to integrate.

Thus, despite the challenges of embedding appropriate, professional expertise into frontline clinical work, there is much to be positive about in the future. The rapid response to the coronavirus disease of 2019 (COVID-19) pandemic has demonstrated this.

## A Timely Example: Human Factors and COVID-19

The spread of COVID-19 exposed a range of challenges with the development, design, dissemination, education, assurance and integration of safe, effective healthcare delivery for a range of clinical contexts. This required substantial adaptation based on complex trade-off decisions between care demands, resources, risks and finances, often without clear authoritative evidence, but from continually evolving information. This can be seen as a ‘microsystems’ problem, where training based on the availability of specific personal protective equipment (PPE) required day-to-day adaptation by staff, based on varying threats and availability; and it can be seen as ‘macrosystems’ issue demonstrating close coupling of acute health, population health, society, commerce and politics.

At a microlevel, the availability of embedded practitioners in acute care demonstrated a range of ways in which HF expertise can be rapidly applied to help clinicians deal with the problem. It was possible to quickly disseminate this information via the HFTH professional network. This work has included training in the use of PPE; designing icons to help with donning and doffing; working to support staff new to intensive care unit through decision support tools; prone-positioning protocols and checklists; COVID-19-specific daily briefing checklists; supporting virtual visits; developing transportation plans; limiting exposure risks from suspected or confirmed patients and developing new protocols for emergency responses. The UK CIEHF rapidly produced guidance on the design of ventilators and the design of work procedures for care teams in all sectors (https://covid19.ergonomics.org.uk/). Several professionals have taken key leadership roles in the responses in their organizations. Others were invited to be involved, including, importantly, in field hospitals where an influx of excess ventilated patients were/are expected. In most cases, it required the HF professional to actively insert themselves into the work, where their perspectives were valued.

At a macrolevel, practices contributed both to the adaptive flexibility required for resilient systems (for example, by taking the load associated with protocol designs off already stretched clinicians), and to the wider understanding of how resilience can be built into future healthcare systems. This includes information sharing; risk perceptions; risk, cost and throughput trade-offs; the need for adaptive skills rather than invariant process following; cross-training; planning for adaptability; and even the use of social media in the absence of other clinical guidance. Indeed, this demonstrated exactly why the linear deterministic view of healthcare systems—where tasks are standardized, variability is controlled, and deviations are ‘errors’—is a deleterious over-simplification of healthcare work, even in otherwise ‘normal’ function. It is people that hold complex systems together; and the goal of is to understand and support the people working in the system to be adaptable and effective, rather than seeing them as a hazard [44].

In short, COVID-19 demonstrates that the ‘new normal’ has benefitted hugely from embedded HF expertise, but the key question is how to build on and sustain this welcome development beyond a pandemic situation.

## The Frontier: Embedding High Quality Human Factors Practice in Healthcare

The ultimate goal of everyone involved in the application of HF principles in healthcare should be to improve the care we deliver to our patients and the working lives of our healthcare professionals. We need to understand and accept that there are multiple routes in, each with different requirements, and each need to be addressed with appropriate professional and practice support. Areas where clinicians and others have applied HF techniques have been most notably in teamwork and teamwork training, simulation training, checklists, education and training, hazard identification and risk assessment, safety culture measurement and discussion and team-based learning from events (Table 1 contains links to some practical resources in these areas). However, a focus on these particular interventions is also symptomatic of the limited nature of application and the persistence of debunked safety science myths. HF professionals, on the other hand, have often struggled to bring their expertise to bear for meaningful clinical benefit. However, organizational, financial and evidence-based pressures are bringing about positive practice changes that are enabling embedded HF professional practice and closer collaboration with clinical professionals, who now have access to appropriate professional certification and supportive professional networks dedicated to the application of healthcare HF.

**Table 1:** Example resources for embedded HF practice

HF topic	Resource
Professional Organizations	Chartered Institute of Ergonomics and Human Factors (UK). www.ergonomics.org.uk Human Factors and Ergonomics Society (USA). www.hfes.org Healthcare Technical Group. www.hctg.hfes.org Human Factors and Ergonomics Society of Australia. www.ergonomics.org.au International Ergonomics Association. www.iea.cc Resilience Engineering Association. www.resilience-engineering-association.org
Clinical Support Networks	Clinical Human Factors Group. www.chfg.org Human Factors Transforming Healthcare Network. www.hfthnetwork.org Solutions for Patient Safety. www.solutionsforpatientsafety.org
Embedding HF in health and social care	Twelve tips for HF principles in healthcare education. https://bit.ly/33ORc8B Human factors in healthcare white paper. https://bit.ly/3kySr1R
Systems Thinking	Safety Culture Discussion Cards. https://bit.ly/3ivAQG5 Systems Thinking for Everyday Work. https://bit.ly/2DLB6C2 From Safety I to Safety II: A White Paper. https://bit.ly/2XRWoVt Learning from Adverse Events. https://bit.ly/3gOWAfO Design of Work Procedures. https://bit.ly/2FiBOaF TeamSTEPPS. https://bit.ly/2PWk5rD
COVID-19 HF Resources	Human Factors Response. covid19.ergonomics.org.uk COVID Risk Assessment Tools. https://bit.ly/2ChoHoM Creating a Safe Workplace. https://bit.ly/3kzQmTk

Clinical systems offer vast potential for both specific improvement for patient and staff benefit, and as a natural laboratory for system safety methodological and theoretical innovation. The interaction of HF and clinical work has already changed both professions. Healthcare is at the forefront of HF frontiers; and HF is at the forefront of clinical frontiers. This is an exciting possibility that everyone involved is keen to realize.

## Funding

The papers were funded by ISQua.
